# Understanding the role of osteoarthrosis on electromyographic activity of masticatory muscles and quality of life

**DOI:** 10.4317/jced.56582

**Published:** 2020-04-01

**Authors:** Mariah Righetti, Oswaldo Taube, Marcelo Palinkas, Lígia Gonçalves, Fernanda Rufato, Veridiana Arnoni, Nayara da Silva, Simone Regalo, Selma Siessere

**Affiliations:** 1 DDS. Department of Basic and Oral Pathology, School of Dentistry of Ribeirão Preto, University of São Paulo, Brazil; 2 DDS, Professor. UNIFAFIBE University Center, Bebedouro, São Paulo, Brazil; 3 DDS, PhD, Professor. Department of Basic and Oral Pathology, School of Dentistry of Ribeirão Preto, University of São Paulo; Faculty Anhanguera, Ribeirão Preto and National Institute and Technology - Translational Medicine (INCT.TM), São Paulo, Brazil; 4 DDS, PhD, Professor. Department of Basic and Oral Pathology, School of Dentistry of Ribeirão Preto, University of São Paulo; National Institute and Technology - Translational Medicine (INCT.TM), São Paulo, Brazil

## Abstract

**Background:**

Osteoarthrosis is a severe, evolutionary, chronic, and limiting disease that influences on quality of life, as it affects synovial joints and promoted degradation of hyaline articular cartilage. We sought to determine if electromyographic activity of the masticatory muscles and quality of life are negatively impacted by osteoarthrosis.

**Material and Methods:**

A sample of 72 participants between 40 and 70 years old diagnosed with osteoarthrosis were selected. Forty-eight participants met the inclusion criteria of this study and were divided into two distinct groups: with osteoarthrosis (n=24) and without osteoarthrosis (n=24). Electromyographic activity of the masseter and temporalis muscles (mandibular rest, right laterality, left laterality, protrusion, and dental clenching in maximal voluntary contraction) and quality of life measurements were used. Electromyographic activity was used to analyze muscle activation patterns. OHIP-14Br and SF-36 questionnaires determined the quality of life.

**Results:**

The participants with osteoarthrosis presented significant greater electromyographic activity (*p*≤0.05) at rest for the right temporal muscle (*p*=0.04), maximum voluntary contraction for the left masseter muscle (*P*=0.04), repercussions of oral conditions on quality of life by the sum of OHIP-14 (*p*=0.002), and a statistically significant difference was found in all subgroups of the SF-36 scale between the subjects with and without osteoarthrosis (*p*≤0.001).

**Conclusions:**

People with osteoarthrosis have changes in electromyographic activity of the masticatory muscles and quality of life compared to healthy participants.

** Key words:**Electromyography, quality of life, bone diseases.

## Introduction

In the group of musculoskeletal diseases, osteoarthrosis is a chronic, evolutionary, painful, and limiting degenerative disease involving the articular cartilage, surrounding tissues, and subchondral bone, and is one of the main osteoarticular diseases due to senescence and senility (1,2). This disease weakens millions of people worldwide, affecting the population over 60 years (3), and the number of cases of this disease is estimated to double by 2020 (4).

Possible joint destruction may affect essential functions in the ability of the human body to move, as well as involve skeletal striated muscles and adjacent structures (5,6). The possible involvement of the temporomandibular joint by osteoarthrosis is a worrying factor, since this joint is associated with the dental arches and the manner in which their occlusal, tongue, and muscle relationships all work together (7,8).

As in other joints, the temporomandibular joint impairment due to osteoarthrosis leads to connective and bone tissue degradation with a tendency to establish ankylosis, which could determine impairments in the stomatognathic system function and quality of life (9). However, the relationship between rheumatic diseases and the stomatognathic system still remains uncertain because there are no studies in the international literature demonstrating the effects of osteoarthrosis on the action potentials of masticatory muscle fibers.

Thus, a better understanding of how masticatory muscles behave in relation to osteoarthrosis would be important information to cautiously formulate functional rehabilitation treatments by health professionals.

Therefore, we sought to determine if electromyographic activity of the masticatory muscles and quality of life are negatively impacted by osteoarthrosis.

Material and Methods

The case-control study was conducted with the Research Ethics Committee approval (protocol N. 55505316.8.0000.5419) and followed policy for the protection of human participants. Prior to study participation, all participants gave their written informed consent.

-Participants and Characteristics

The post hoc sample size was calculated based on an error of 10% (α = 0.10), and a power test of 85% for the main result of the electromyographic activity in the condition of raisin chewing (average values of the right temporal muscle). The value for the group with osteoarthrosis was 1.27 (0.86), and for the control group, the value was 0.81 (0.43), and the effect size was 0.67. The minimum sample size was 48 subjects (24 for each group). The sample size was calculated using the free-access software program G* Power version 3.1.9.2 (Franz Faul, Kiel University, Kiel, Germany).

These participants were selected after anamnesis, and dental examination was performed by a trained operator to establish legitimacy. A total of 72 participants of both genders, aged between 40 and 70 years, were selected. Per the inclusion and exclusion criteria, a final 24 participants were selected for the group with osteoarthrosis (mean ± s.d.: 53.6 ± 1.6 years old and 28.61 ± 0.98 kg/m2). Participants with Angle Class I contact pattern in maximum intercuspal position with tooth to two tooth occlusion and presence of all permanent teeth (except third molars) were selected. The disease-free group (mean ± s.d.: 52.3 ± 1.7 years old and 28.16 ± 0.78 kg/m2; n=24) was composed of dentate individuals without temporomandibular dysfunction (RDC/TMD) who were age-, gender-, weight-, and height-matched with subjects in the osteoarthrosis group. The exclusion criteria for study and control groups involved the absence of dental elements (upper and lower), temporomandibular dysfunction, the presence of dental restorations with risk of fractures, and the use of anti-inflammatories, analgesics, or muscle relaxants that could interfere in neuromuscular physiology. Participants with osteoarthrosis were diagnosed by a rheumatologist following the guidelines of the American College of Rheumatology, according to the following criteria: hip, knee and hand joint pain (10).

-Analysis of electromyographic activity

The electromyographic tests were performed to determine the activation pattern of the masseter and temporalis muscles using Trigno wireless electromyography equipment (Delsys Inc., Boston, MA, USA). The sensors’ positions were determined by palpation during muscle contraction and were fixed with an adhesive tape with the longest extension perpendicular to muscle fibers (11). Before positioning of the sensors, the skin went through an alcohol sanitization process to reduce the impedance, and the sensors were fixed after a few minutes of the procedure (12).

Instructions and explanations were given, which requested calm and tranquility during the exam. During the tests, the subjects sat in a comforTable office chair while maintaining their upright posture, with feet flat on the floor and arms resting on their legs. We analyzed the masseter and temporalis muscles in both sides; for the static evaluation, the participants were evaluated during rest, protrusion, laterality movements (right and left), and maximal voluntary dental clenching for 4 s. Additionally, the maximal voluntary contraction with inert material was recorded for 4 s, so that the electromyography samples could be normalized (13). The inert material consisted of a paraffin sheet (Parafilm M, Pechiney Plastic Packaging, Batavia, IL, USA) inserted between the occlusal faces of the upper and lower first molars on the right and left sides of the dental arch.

The gross electromyographic signal (microvolts/second) was applied to derive electromyographic amplitude values obtained by calculating the square root mean used for mandibular tasks.

-Quality of Life Assessment

The Oral Health Impact Profile 14 Brazil (OHIP - 14Br) questionnaire was used to analyze participants’ perceptions of the social impact of oral cavity disorders and health over the past 12 months (14). The OHIP -14Br consists of 14 questions distributed in seven dimensions: functional limitation, physical pain, psychological discomfort, physical limitation, psychological limitation, social limitation, and disability (15-17). The Medical Outcomes Study 36 - Item short - Form Health Survey (SF-36) was used to assess the participant’s health (18). The SF-36 consists of 36 questions, which are divided into eight scales or domains: functional capacity, physical aspects, pain, general health, vitality, social aspects, emotional aspects, and mental health (19). The Likert scale was used to analyze the results of the participants’ questions, so that the lower the scale value, the better the quality of life.

-Method Error

For the reliability of the results, Dahlberg’s formula was used to demonstrate casual error in this study. Measurements of normalized electromyographic activity were calculated using the recordings of five participants and were obtained during two different sessions with an interval of seven days. A small difference was observed in measurements between the first and second sessions on electromyographic activity (3.74%).

-Statistical analysis

Data on normalized electromyographic and quality of life data were submitted to statistical analysis using IBM SPSS Statistics for Windows version 22.0 (IBM SPSS, IBM Corp., Armonk, NY, USA). Results were obtained by descriptive analysis (mean and standard error) for each variable. Significance was accepted at *p*≤ 0.05 (student’s t-test).

## Results

The data presented in Table 1 showed significantly greater electromyographic activity (*p*≤ 0.05) at rest for the right temporal muscle (*p*=0.04) and maximum voluntary contraction for the left masseter muscle (*p*=0.04) in the osteoarthrosis group.

**Table 1:**
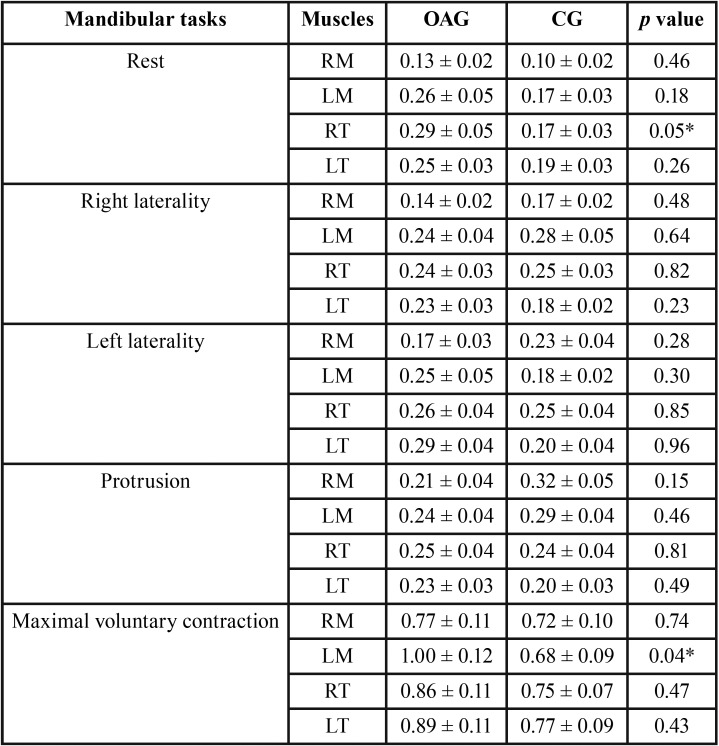
Means, standard errors (±) and statistical significance (*p*≤0.05*) of the normalized electromyographic data averages of the right masseter (RM), left masseter (LM), right temporal (RT) and left temporal (LT) for with osteoarthrosis (OAG) and disease-free (CG) groups.in the mandibular tasks.

The results of the OHIP-14Br questionnaire between groups are presented in Table 2. Higher scores were observed in the osteoarthrosis group vs. the without the disease group. This significant difference (*p*≤ 0.05) was more evident when OHIP-14 issues were grouped in domains characterized by functional impairment, psychological discomfort, physical impairment, psychological impairment, and disability (*p*≤0.05). Repercussions of oral conditions on quality of life by the sum of OHIP-14 (*p*=0.002).

**Table 2:**
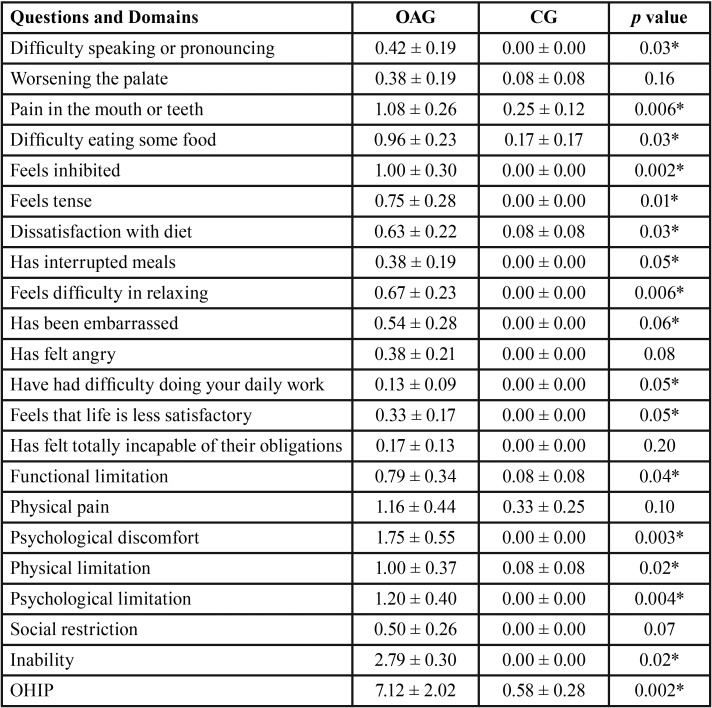
Means, standard errors (±) and statistical significance (*p*≤0.05*) of the OHIP-14 questions and domains for with osteoarthrosis (OAG) and disease-free (CG) groups.

In terms of assessment scales, a significant difference was found in all subgroups of the SF-36 scale between the participants with and without osteoarthrosis (*p*≤0.001) (Table 3).

**Table 3:**
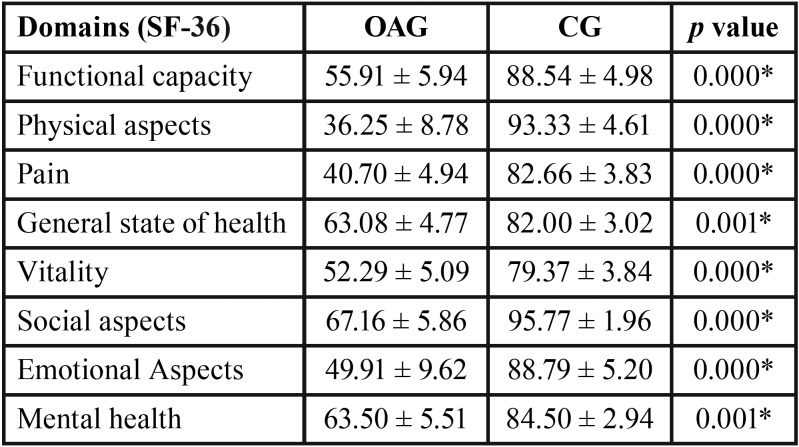
Means, standard errors (±) and statistical significance (*p*≤0.05*) of the SF-36 domains for with osteoarthrosis (OAG) and disease-free (CG) groups.

## Discussion

The findings of the present study showed that by analyzing the electromyographic activity at mandibular rest, the participants of the osteoarthrosis group showed greater activities in the masseter and temporalis muscles with a significant difference in the left temporal muscle. Our results are different from the literature, which shows that in the mandibular rest condition, the myoelectric activity is minimal or nonexistent and shows no motor unit contraction (20).

In the maximal voluntary contraction dental clenching, the left masseter muscle of the osteoarthrosis group presented significantly greater values compared to the group without the disease. When analyzing the osteoarthrosis group, there was a break in the neuroanatomic pattern, where the temporalis muscles were more active than the masseter muscles (21). The masseter muscle is known to be a force muscle, while the temporal muscle is related to the speed of movement (22,23).

The change in the electromyographic activity of the masticatory muscles in isometric contraction originates from the central nervous system and may be correlated with psychological changes (21,24). Our results showed overload in the left masseter muscle in the osteoarthrosis group.

In the right laterality, greater electromyographic means were observed in the left temporal muscle for the osteoarthrosis group. In the evaluation of the other masticatory muscles, the opposite was observed, but without significant difference. When comparing the groups in the left laterality, it was evident that there was a difference in the neuroanatomical pattern in this excursive movement, in which it was expected that the left temporal muscle should show greater activation than the right temporal muscle. Moreover, the right masseter muscle should be more activated than the left masseter muscle (25).

We observed that the osteoarthrosis group presented greater electromyographic activity of the left masseter muscle during the left laterality movement in relation to the right masseter muscle, which departs from the standard established in the international literature (11,21). One of the main clinical signs of dysfunctional problems with the masticatory system is the limitation of mandibular movements (26). The hypothesis to explain the different patterns observed in the mandibular laterality movement could be related to unilateral involvement of temporomandibular joints in participants with osteoarthrosis. In this study, the involvement of the temporomandibular joints by osteoarthrosis was not evaluated. 

In the protrusion, although the comparison between the groups did not show significant difference, it was possible to observe through analyzing only the osteoarthrosis group higher electromyographic means for the right temporal muscle when compared to the masseter muscles. The muscle pattern for maintaining the protrusion movement is known to be a greater activation of masseter muscles than temporal muscles (27). Thus, during this postural condition, it was again observed that the patterns presented by the osteoarthrosis group were different from those found for participants without the disease.

This different pattern that was found in participants with osteoarthrosis may be associated with greater horizontal condyle angulation in relation to this disease, as demonstrated by Lee *et al.* (27) through computed tomography exams. According to these authors, the greater condyle angulation precedes the appearance of osteoarthrosis (28).

In this study, the OHIP-14Br questionnaire was used to assess the impact of oral health conditions on the quality of life of participants with osteoarthrosis and without osteoarthrosis. Our results allowed us to identify significant negative impacts of oral conditions on the quality of life of participants with chronic degenerative disease, with mean OHIP-14 summation values of 7.12 (± 2.02) for the group with osteoarthrosis and 0.58 (± 0.28) and for the group without the disease.

Our study demonstrated that osteoarthrosis can compromise the quality of life, both in its physical aspect, as well as in the psychological and social aspects. Our results are in agreement with those of Su *et al.* (29) who evaluated a total of 541 participants with a confirmed diagnosis of osteoarthrosis (34 men and 407 women) and found total OHIP-14 Br score values of 16.10, which was significantly higher than the values found for participants without the disease (11.98), demonstrating that the oral condition was negatively influenced by osteoarthrosis in the temporomandibular joint.

Another method used in this study was the SF-36 questionnaire that assessed quality of life through specific domains with a significant difference in all subgroups of the SF-36 scale between the participants with and without osteoarthrosis. The group with osteoarthrosis had a worse quality of life. Quality of life is directly related to health; therefore, actions that promote well-being and health should be based on prevention of preventable diseases, postponement of diseases, early care, and rehabilitation of diseases.

Thus, to put into practice all the actions necessary for a healthy and quality of life in an aging population, it is necessary to rethink and redesign the care for the elderly, focusing on the individual and their particularities (30).

This study had some limitations, such as not using computed tomography to show the involvement of the temporomandibular joint, as well as the other structural components, by osteoarthrosis. We mentioned computed tomography because the use of panoramic radiographs and magnetic resonances may result in false-negative findings, i.e., they do not allow for diagnosis even when the disease is present (31).

Another important factor mentioned in this study is that participants were in the early stage of osteoarthrosis. Possibly, there may be a greater involvement in the joints with the evolution of the disease, which greatly reflects on postural conditions and impairment of quality of life. Thus, further studies are suggested over time to verify this hypothesis.

## Conclusions

The authors of this study suggest that people with osteoarthrosis have functional changes in the stomatognathic system, especially in normalized electromyographic activity of the masseter and temporal muscles, with a negative impact on the quality of life.
